# Nurse-Delivered Cognitive Behavioral Therapy for Adherence and Depression Among People Living With HIV (the Ziphamandla Study): Protocol for a Randomized Controlled Trial

**DOI:** 10.2196/14200

**Published:** 2020-02-03

**Authors:** John A Joska, Lena S Andersen, Rosana Smith-Alvarez, Jessica Magidson, Jasper S Lee, Conall O’Cleirigh, Steven A Safren

**Affiliations:** 1 HIV Mental Health Research Unit, Neuroscience Institute Department of Psychiatry and Mental Health University of Cape Town Cape Town South Africa; 2 Department of Psychology University of Miami Coral Gables, FL United States; 3 Department of Psychology University of Maryland College Park, MD United States; 4 Massachusetts General Hospital Harvard Medical School Boston, MA United States

**Keywords:** major depressive disorder, cognitive behavioral therapy, HIV, medication adherence, integrated treatment, task shifting

## Abstract

**Background:**

There is an unmet need to develop effective, feasible, and scalable interventions for poor adherence and depression in persons living with HIV in low- and middle-income countries (LMIC).

**Objective:**

This study aims to investigate the effectiveness of a nurse-delivered cognitive behavioral therapy (CBT) intervention for adherence and depression (CBT-AD) among persons living with HIV who are failing first-line antiretroviral therapy (ART) in Cape Town, South Africa.

**Methods:**

This study is a 2-arm randomized controlled trial of CBT-AD integrated into the HIV primary care setting in South Africa. A total of 160 participants who did not achieve viral suppression from their first-line ART and have a unipolar depressive mood disorder will be randomized to receive either 8 sessions of CBT-AD or enhanced treatment as usual. Participants will be assessed for major depressive disorder using the Mini International Neuropsychiatric Interview at baseline and 4, 8, and 12 months. The primary outcomes are depression on the Hamilton Depression Scale (HAM-D; as assessed by a blinded assessor) at the 4-month assessment and changes in ART adherence (assessed via real-time, electronic monitoring with Wisepill) between baseline and the 4-month assessment. Secondary outcomes are HIV viral load and CD4 cell count at the 12-month assessment as well as ART adherence (Wisepill) and depression (HAM-D) over follow-up (4-, 8-, and 12-month assessments).

**Results:**

The trial commenced in August 2015 and recruitment began in July 2016. Enrollment was completed in June 2019.

**Conclusions:**

Results of this study will inform whether an existing intervention (CBT-AD) can be effectively administered in LMIC by nurses with training and ongoing supervision. This will present unique opportunities to further explore the scale-up of a behavioral intervention to enhance ART adherence among persons living with HIV with major depression in a high-prevalence setting, to move toward achieving The Joint United Nations Programme on HIV/AIDS 90-90-90 goals.

**Trial Registration:**

ClincialTrials.gov NCT02696824; https://clinicaltrials.gov/ct2/show/NCT02696824

**International Registered Report Identifier (IRRID):**

DERR1-10.2196/14200

## Introduction

### Background

The global HIV epidemic has disproportionately affected low- and middle-income countries (LMIC) and Southern Africa in particular [1]. In this region, South Africa has the highest rate of HIV infection and the highest number of persons living with HIV (PLWH) [1]. Coupled to this, it also has the largest antiretroviral therapy (ART) treatment program [2]. Successful management of the epidemic in these high-prevalence settings requires effective interventions across the care cascade. In 2017, it was reported that only approximately 30% of HIV-infected individuals in South Africa had achieved viral suppression [3]. One key aspect of effective viral suppression are behavioral adherence factors, including the management of mental disorders, such as major depressive disorder (MDD).

Depressive disorders are highly prevalent among South Africans in general, with the lifetime prevalence of MDD being 9.8%, whereas PLWH are at increased risk of developing a depressive disorder [4]. Rates of 10% of major depression and nearly 30% of minor depression have been noted in PLWH in South Africa [5]. Mental health services are not geared to address depressive disorders in general in PLWH in primary health settings, resulting in a large treatment gap—only 25% of persons affected by a common mental disorder ever obtain treatment [6]. Reasons for this treatment gap include the burden of large patient numbers and few mental health professionals, limited treatment options, and a general focus on hospital-based services as opposed to clinic- or community-based ones where the focus is often on severe mental disorders [7-9]. Counseling and psychotherapy for depressive disorders are not readily available in primary health care because of constraints in skills and resources [10,11]. As qualified psychologists are in short supply in South Africa, the use of mental health nurses, who staff the community mental health clinics, represents an opportunity to transfer critical cognitive behavioral therapy for adherence and depression (CBT-AD) skills more widely.

The impact of untreated MDD includes several adverse health outcomes, such as HIV disease progression and mortality [12], as well as reduced quality of life, disability, and increased time out of role [13,14]. A significant mediator of these outcomes in the context of untreated MDD is poor medication adherence [15]. PLWH in South Africa face several psychosocial challenges known to increase the risk for depressive disorders [16] and also several stressors unique to living with HIV, such as stigma, loss, and the challenges of living with a chronic disease [17,18]. Strategies to improve depression and coping with living with HIV within primary health care are, therefore, imperative.

In LMIC, there is a lack of consensus on how best to provide mental health care for PLWH [19,20]. Owing to resource constraints, such as a lack of qualified clinical psychologists to deliver evidence-based psychosocial treatments and a lack of psychiatrists to evaluate and further treat by utilizing the available medications in LMIC, health systems have utilized task-sharing approaches, with varied success [21,22]. The delivery of evidence-based psychotherapy for a combination of MDD and adherence difficulties by paraprofessionals would represent a significant step forward in addressing this issue among PLWH [23].

CBT is an effective treatment for MDD, with a strong evidence base [24]. In the context of MDD in chronic disease care, there are data to support its use to improve both MDD and adherence [25-27]. There is growing evidence that CBT for both adherence and MDD (ie, CBT-AD) might be effective in LMIC and in South Africa in particular. In an open case series of 14 sessions of CBT for depression in HIV clinics in Cape Town [28], 6 participants experienced a marked reduction in depressive symptoms during the course of treatment. This study established initial feasibility and acceptability for CBT for depression applied to PLWH in South Africa with a CBT psychologist as the interventionist. A further pilot study of 14 participants evaluated a shortened, nurse-delivered, integrated CBT-AD intervention adapted to the South African context [29]. Although improvements in adherence and mood were robust, there were challenges in provider fidelity to the intervention, which required intensive training and supervision. Further work to develop an effective, yet scalable, CBT-AD intervention is, therefore, necessary. In addition to the key issues of adherence and remission of depression, the issue of task-sharing of the intervention to nonpsychologists needs to be explored.

### Trial Objectives

The goal of this trial is to investigate whether CBT-AD administered by nurses is effective for reducing depression and improving ART adherence among PLWH with adherence difficulties and MDD in primary health care in Cape Town, South Africa.

The primary objective is to compare the effectiveness of an isiXhosa-adapted, nurse-delivered CBT-AD intervention integrated into the HIV care setting in individuals who are failing, or have failed, first-line ART. The CBT-AD treatment is being compared with enhanced usual care (enhanced treatment as usual [ETAU]) at 12 months. ETAU consists of participants’ medical providers being furnished with a referral letter indicating the psychiatric conditions they meet diagnostic criteria for including MDD. Primary study end points include depression scores on the Hamilton Depression Scale (HAM-D; as assessed by a blinded assessor) at the 4-month assessment and changes in adherence scores (assessed via the electronic Wisepill device) between baseline and the 4-month assessment.

Secondary outcomes include HIV viral load and CD4 cell count at the 12-month assessment as well as adherence (Wisepill) and depression (HAM-D) over follow-up (4-, 8-, and 12-month assessments).

## Methods

### Trial Design

**Figure 1:**
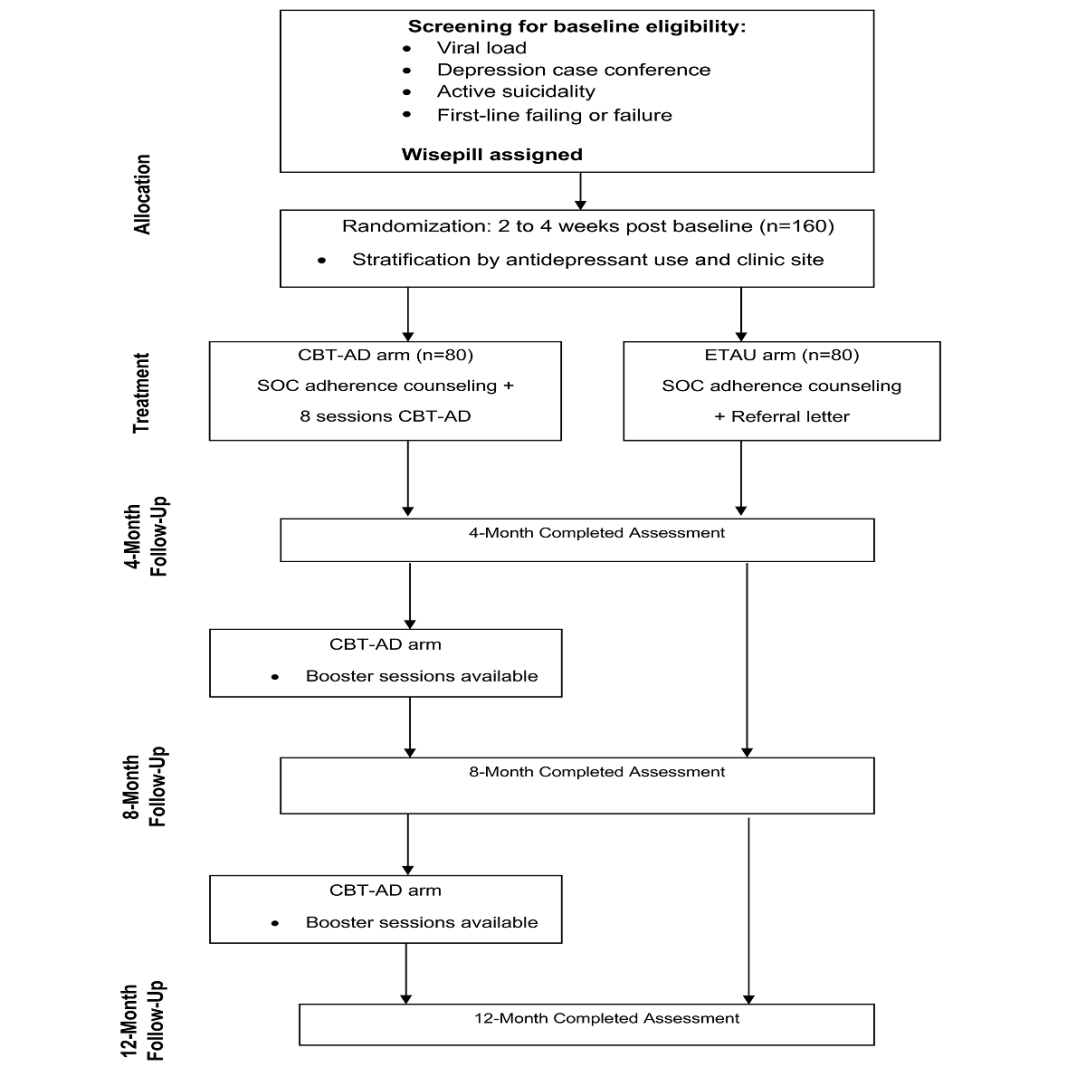
Consolidated standards of reporting trials diagram. CBT-AD: cognitive behavioral therapy for adherence and depression; ETAU: enhanced treatment as usual; SOC: standard of care; 4M: 4-month; 8M: 8-month; 12M: 12-month.

Project Ziphamandla (meaning *to be empowered* in isiXhosa) is a 2-arm, randomized controlled trial comparing CBT-AD with ETAU among PLWH who are failing, or have failed, first-line ART (see Consolidated Standards of Reporting Trials diagram in Figure 1).

Before randomization to treatment condition, participants complete a diagnostic assessment, clinician-rated measures of depression, and a self-report psychosocial assessment battery. We aim to randomize 160 participants to either CBT-AD (8 sessions) or ETAU. For participants in both conditions, treatment providers receive letters summarizing the results of the participants’ diagnostic assessments. At this point, their treatment providers may further diagnose or treat (with antidepressants) MDD in the participants. Randomization follows approximately 1 month after the baseline assessment, so that stratification can occur by those who may or may not have commenced antidepressants as part of their care, following this letter. The 1-month period from baseline to randomization also allows for a baseline assessment of ART adherence using Wisepill real-time electronic adherence monitoring. For participants in both arms, there are 4 major study assessment points: baseline, post intervention (4-month), 8-month, and 12-month.

### Study Sites and Participants

#### Recruitment

All participants are enrolled at primary HIV care clinics located in periurban areas of Cape Town. Potential participants are identified by their HIV primary care clinic as not responding to their current antiretroviral (ARV) treatment regimen (defined per local clinic standards as viral load >400 copies/mL). Research assistants (RAs) and other study staff situated at the primary care clinics work with staff in the HIV clinics to identify potential participants as they wait for their appointments. RAs are isiXhosa-speaking staff recruited locally for the study based on their research and clinical experience. They are extensively trained in the study protocol and supervised weekly by the project director who is a PhD-level clinical psychologist. The team also makes use of recruitment materials (eg, fliers) to describe that the study is for individuals who are managing depression and are not responding to their current ARV treatment regimen. Potential participants are screened using the MDD module of the Mini International Neuropsychiatric Interview (MINI) [30]. If individuals screen positive for MDD, but they do not have a recent (1 month) viral load, they are asked to undergo a blood draw to determine if their viral load is unsuppressed (ie, >400 copies/mL).

To be eligible for the study, participants must (1) be HIV seropositive, (2) have a current diagnosis of MDD (meets threshold for diagnostic severity based on the MINI), and (3) not have attained viral suppression from first-line ART. Participants are excluded if they (1) are unable or unwilling to provide informed consent, (2) have active untreated major mental illness (untreated psychosis or mania or high suicide risk) that would interfere with CBT-AD, (3) have received CBT for depression, or (d) are aged <18 years.

### Procedures and Study Assessments

#### Baseline

At baseline, participants complete the informed consent process and sign the release of information form to allow access to their medical record for most recent viral load and CD4 and current ART regimen. The nurse interventionists conduct the clinical assessment and administer the HAM-D and the remaining MINI modules. The RAs administer the battery of psychosocial assessments and other self-report questionnaires. The study team, including 2 clinical psychologists, meets to discuss each case and confirm that participants meet diagnostic criteria for MDD.

Participants also provide consent to allow the study staff to contact them for up to 1 year after completion of the study (ie, 1 year after the 12-month follow-up visit) for additional study purposes. The team makes use of mobile phones as well as meets participants at clinics when they attend other clinic appointments, as phone numbers do change frequently. In addition, participants also consent to allow the study to continue to access medical records at their clinic (eg, viral load and CD4 cell count) for a period of 5 years following completion of the study. We anticipate that approximately 1000 prospective participants need to be screened and consented to randomize the sample of 160 fully eligible individuals.

#### Randomization

Approximately 1 month after the initial baseline assessment, participants return for their randomization visit. At this visit, participants are randomly assigned to either CBT-AD or ETAU. Randomization is determined by a computer-generated chart, using the Research Electronic Data Capture (REDCap) randomization module [31]. Study staff enter stratification information (specifically, antidepressant use and site) and click randomize. Antidepressant use is confirmed by paper medical record review. Once the condition has been assigned in REDCap, this field is locked and cannot be changed.

As the study team sends a letter to HIV providers documenting the participants’ depression, regardless of study arm, participants are given the opportunity to start using antidepressant medication outside of the study if they are not already using them. Therefore, at the randomization visit, we stratify randomization by whether or not the participant is taking an antidepressant (2 levels). We are also stratifying by which study site the participant is seen at (2 levels). Note our Data Safety and Monitoring Board (DSMB) reviews baseline data annually for adequacy of randomization.

#### Retention

The RAs track participant retention, which is reviewed weekly by the on-site project director. Procedures to maximize retention include reimbursing participants for their transport costs, providing participants with an appointment card, contacting participants to remind them of their scheduled appointments, and offering a R200 grocery voucher to participants who complete the full course of treatment. We also collect extensive locator information (eg, contact information of 2 significant others with whom the participant is in regular contact). We make efforts to maintain contact with individuals who move to a nonstudy site for their HIV care but are still willing to complete follow-up.

Following baseline, there are 3 further major assessment visits: post intervention (4 months), 8 months, and 12 months. At the 4-, 8-, and 12-month follow-up assessments, an independent assessor (IA), blind to study condition, repeats the clinician-rated assessments and administers the self-report measures to the participants. Bloods are drawn to determine viral load and CD count (unless available through medical chart proximal to the visit). The follow-up assessments occur at the study sites. However, in the event that a participant is unable to attend the study sites, the assessment may be conducted telephonically and arrangements can be made to have bloods drawn at the closest local facility.

All participants receive standard HIV and ART adherence counseling, comprising 3 to 4 sessions as part of standard of care at the clinic. Participants randomized to the integrated intervention (CBT-AD) receive their first intervention visit following randomization and receive up to 8 intervention sessions (plus optional booster sessions).

### Interventions

#### Intervention Training

Before starting the study, nurse interventionists completed a weeklong training in the intervention that was conducted by the US and South African investigators. The South African investigators then provided further training until the nurses were deemed competent to begin administering the intervention. Since the nurses started administering the intervention to participants, the project director and the other South Africa–based clinical psychologist provide weekly supervision for an hour with support from the US investigators when needed. Moreover, 1 therapy session from each interventionist is orally translated by the RAs during the week it occurred and is listened to by the clinical psychologists in preparation for supervision. Approximately yearly, interventionists have booster training with the US and South African investigators to prevent drift and enhance fidelity and competence to the protocol.

#### Standard of Care—Enhanced Treatment as Usual

The clinic nurse provides feedback to the participant and to their clinic doctor (via a letter) about their MDD diagnosis. In addition, all participants undergo standard of care adherence counseling in the clinic, which may include several sessions with a clinic counselor. The letter specifically states that they are not restricted in terms of referral or treatment of MDD for their patient with respect to antidepressant medications or other interventions available.

#### Treatment Intervention—Cognitive Behavioral Therapy Intervention for Adherence and Depression

The intervention is the specific version that we have adapted to this setting based on a series of pilot studies [29]. Adherence and self-care issues are incorporated into each session. In early sessions, we employ motivational interviewing and psychoeducation to establish confidence in, and credibility of, the treatment and to increase motivation for retention in the study. In addition, information specific to the participant is gathered during this interactive process, with careful attention to the role of specific life stressors that may influence case conceptualization. After session 1, which focuses on adherence counseling, at the start of each session, the nurse interventionist discusses adherence and depressed mood over the past week, reviews the results from the Wisepill as a graph depicting actual adherence in the past week, and discusses any adherence issues. This review allows for a discussion of progress and consideration of any problem solving or enhancements to the homework and coping plan assigned in previous sessions. To integrate CBT for depression with adherence skills, participants review adherence problem solving before starting the weekly CBT-AD sessions that focus on both depression and adherence/self-care. For this study, to maximize fidelity to the intervention, it is delivered in the form of a flip-book that the interventionist and participant both see, with additional notes on one side for the interventionist and visual depictions for the participant.

#### Session 1: Life-Steps

Life-Steps [32,33] is a single-session intervention based on general principles of CBT as well as more specific principles of problem-solving therapy. The adapted Life-Steps incorporates 13 informational, problem-solving, and cognitive behavioral components: (1) psychoeducation, (2) transportation to appointments, (3) obtaining medications, (4) formulating a daily medication schedule, (5) plan for storing medications, (6) plan for obtaining medications when away from home, (7) identifying social supports, (8) identify motivation for adherence and create association with reminders, (9) plan for coping with medication side effects, (10) communication with treatment team, (11) plan for taking medication when using substances, (12) responses to slips in adherence, and (13) review of plans. In each step, the participant and clinician define the problem, generate alternative solutions, make decisions about the alternatives, determine the optimal solution, and develop an action plan to implement that solution.

#### Session 2: Psychoeducation and Motivational Interviewing

A major focus of the first session involves gathering and presenting information in a way that promotes credibility and confidence in the treatment for participants [34]. Following principles of culturally informed functional assessment and case conceptualization [35-37], the nurse interventionist and the participant go over the CBT model of depression and, using motivational interviewing, identify specific motivations for behavior change and attempt to resolve participant ambivalence.

#### Sessions 3 and 4: Behavioral Activation

This module addresses depression-related withdrawal from pleasurable activities and social interactions. Participants learn to rate their mood with respect to the activities of their life and learn to systematically program *pleasure-* and *mastery-based* activities back into their lives. This module has been extensively adapted for patients with very limited resources who are living in poverty, including an isiXhosa-translated and locally adapted pleasant events checklist.

#### Sessions 5 and 6: Problem Solving

This involves teaching participants how to define and approach problems; generate and rank order alternative solutions; and implement optimal solutions. Participants are also taught how to break down a complex problem into manageable steps. This approach is used for the treatment of depression [38] and coping with chronic illness [39] and was reviewed positively by patients in our pilot study [29].

#### Session 7: Relaxation Training

Progressive muscle relaxation and diaphragmatic breathing are used to support management of negative mood and stress. Progressive muscle relaxation comprises tensing and relaxing particular muscle groups to learn to distinguish between the feelings of tensed versus relaxed muscles. Ultimately, the goal is to enable the participant to cue their muscles to relax when they start to feel anxious. These relaxation techniques are also widely used in behavioral medicine approaches to managing body pain, headache, and nausea [40], all of which can be side effects of ART and can interfere with adherence.

#### Session 8: Review and Relapse Prevention

One goal of CBT is to transition patients to *be their own therapists* and continue to use the skills after the active intervention ends. Accordingly, this session makes such plans and involves discussing how the participant may continue to cope with emergent life stress.

#### Booster Sessions

After completing the 8 sessions outlined above, participants randomized to the CBT-AD treatment condition also may attend up to 9 optional booster sessions throughout follow-up. Booster sessions comprise topics and skills from the previous 8 sessions to address any additional difficulties with adherence and/or depression, tailored to the individual and based on notes of their sessions.

#### Nurse Interventionist Protocol Integrity

The integrity and feasibility of the specific protocol is assured empirically. All intervention sessions are digitally recorded and subsequently translated into English by the RAs. Each session of 1 hour takes approximately 2 hours to translate. Participants provide consent for this but are not excluded if they refuse this aspect. Monitoring of the intervention takes into account both therapist adherence and competence [41]. We have developed a rating checklist to evaluate the nurse counselors adherence to CBT-AD, including whether the specific treatment components were, in fact, delivered.

### Measures

#### Diagnostic Evaluation

The MINI 7.0 for Diagnostic and Statistical Manual of Mental Disorders-5 is one of the most widely used diagnostic instruments to reliably determine psychiatric disorders in clinical populations [42-45]. The MINI is designed to be used by clinicians but can be administered by nonclinicians with appropriate training and supervision. The recruiters conducting the screening, the nurse interventionists, and the IA received training and weekly supervision on the MINI.

#### Primary Outcomes

##### Depression

The nurse interventionists at baseline and the IA at the follow-up assessments complete the HAM-D [46], one of the most widely used clinical measures of depression in psychiatric research that has strong psychometric reliability and validity. It has also been used in several antidepressant medication trials in South Africa [45,47,48]. Assessors are trained by certified trainers and licensed psychiatrists/psychologists on the study. The self-reported Center for Epidemiologic Studies Depression Scale [49] is also used. This measure has been translated into isiXhosa and used successfully in South Africa.

##### Assessment of Adherence to Antiretroviral Therapy

Wisepill is a real-time, electronic adherence monitoring system that comprises a pillbox container fitted with a global system for mobile communication chip. Using mobile phone technology, the Wisepill transmits a real-time signal to the Wisepill Web server each time the pillbox is opened. The Wisepill Web server can be securely accessed from any computer via the Wisepill Technologies website with the use of a designated log-in and password. It captures real-time data on device openings and, hence, provides the results of weekly assessments for longitudinal modeling. For the purposes of this study, we did not establish a *window* for missed doses; they were just counted as whether or not they opened the device the required number of times (ie, if they opened the device on 2 separate occasions for a twice daily regimen, it would be counted as fully adherent for the day, and if they opened it once for a twice daily regimen, it would be counted as 50% for the day). To ensure the Wisepill device is working properly, we check in with participants at approximately half-way between major study visits (ie, at approximately 2, 6, and 10 months in study participation). In addition, we monitor Wisepill functionality between the baseline visit (when the device is issued) and the randomization visit. To ensure the device is working properly, if no signal is detected from the Wisepill device for 3 or more days in a row within the first week after the device has been issued, we contact the participant to ascertain if they are experiencing any issues with their device. The purpose of these check-ins is solely to enquire about any difficulties using the Wisepill device. The Wisepill Web server also monitors device battery levels, and participants whose devices had low battery are contacted to remind them to charge the Wisepill device.

#### Secondary Outcomes: HIV Viral Load and CD4 Cell Count

Absolute CD4 cell count and number of HIV viral copies per milli liter of blood are extracted from the participants’ medical record at baseline (to assess the first-line treatment failure inclusion criteria) and at 12-month follow-up. This allows for a calculation of the proportion of patients with virologic suppression. Participants who do not have viral load test results within 1 month of the baseline assessment or 4-, 8-, and 12-month follow-up undergo a blood draw for assay using the COBAS AmpliPrep/TaqMan HIV-1 test (range: 20-10,000,000 copies/mL) [50].

#### Psychosocial Self-Report Assessment Battery

Participants complete a demographic questionnaire including items regarding age, sex, sexual orientation, educational history, and employment status. National Institutes of Health (NIH)–defined categories are used to assess participant race and ethnicity, although we anticipate mostly (if not only) isiXhosa-speaking black South African participants. We also obtain data on mode of HIV infection, using a single-item (AIDS Clinical Trials Group) question assessing risk factors for likelihood of means of HIV infection.

#### Medications

During baseline assessment, participants will provide information regarding all of their medications (psychiatric, HIV, and other). When completing assessments at every study visit thereafter, patients are asked to report any changes to their medication regimen. These changes are recorded on adherence questionnaires and cross-checked with clinic chart review. Any initiation or changes to antidepressant medications are tracked across all participants and will be used as covariates in analyses.

### Data Analysis

#### Primary Outcome Measures

The primary outcome analyses compare changes in depression (HAM-D; continuous measure assessed by a blinded assessor) and adherence scores (continuous measure assessed via Wisepill) from baseline to the 4-month assessment.

#### Secondary Outcome Measures

Secondary outcome measures include viral load, analyzed categorically (suppressed vs not) at the 12-month outcome, using <400 copies/mL as suppressed (consistent with recent work analyzing chart-extracted viral load data from the same clinics in Khayelitsha). CD4 is analyzed continuously at the 12-month outcome. In addition, HIV medication adherence and depression scores over time are examined as secondary outcomes using the 4-, 8-, and 12-month assessment time points.

The ultimate data analytic approach will depend on the distribution of the data. We anticipate that we will use generalized linear models), which are estimated using generalized estimating equations with robust standard error estimates to account for repeated measures of the outcome. For the primary outcomes, we will examine adherence scores longitudinally using the Wisepill data and Hamilton scores using the 2 time points (baseline to 4 months) comparing the 2 study arms. For outcomes assessed at the 4 major assessments (ie, baseline and 4, 8, and 12 months), we will use repeated measures analyses. For 12-month viral load, we will test the proportion of those who are suppressed using Fisher exact test. If continuous viral load data are analyzed, we will make log transformations and examine viral load using repeated measures. To reduce bias, the intention-to-treat principle is utilized, where individuals are analyzed according to the condition they were randomized, regardless of their fidelity to that condition.

#### Exploratory Outcomes

We examine the possible effects of alcohol and substance use by means of the World Health Organization’s alcohol, smoking, and substance involvement screening test [51]; the alcohol use disorders identification test [52]; and the MINI [30], and we examine demographic factors as moderators to the effects of the nurse-delivered CBT intervention. For these analyses, we fit interaction terms to the models. We also separately assess effects of the treatment in subgroups defined by these factors. If results indicate that the intervention significantly increases adherence, we then assess the extent to which this effect operates through possible mediators (eg, depression, coping, and social support). In the first set of models, we add the main effects of alcohol (as an example) and time (ie, the different assessment time points), in addition to an intervention by alcohol use interaction and intervention by time interaction. We conduct a product of coefficients test for the effect of each mediator as an intervening variable [53]. Statistical significance of the mediated effect will be determined by the asymmetric distribution of products test. This test is conducted separately for the other mediators.

#### Sample Size Considerations

The study is powered to detect differences in the depression outcome as well as virologic suppression, and accordingly, a sample size of 160 randomized participants is appropriate. Using repeated measurements with a medium effect, we will have almost 90% power to detect a 10% difference in depression with complete retention at randomization, and assuming 20% attrition, we will have 84% power to detect a difference. On the basis of our earlier efficacy trial that was completed when this study was being designed, for those who entered the study with detectable viral load (in this study, all participants entering will have detectable virus), there was a 20% difference in the proportion of those who had undetectable virus at 12-month follow-up: 40% of those in the control condition compared with 60% of those in the experimental condition [27]. On the basis of these effect sizes, for this study, with a sample size of 160, we are powered to detect differences in the secondary binary outcome, virologic suppression, as follows: we will have >90% power to detect at least a 25% difference and >80% power to detect at least a 20% difference in the proportion of those with detectable viral load. These calculations assume a 20% attrition rate that occurs during the active treatment phase of the study, that the proportion of subjects with viral suppression in the usual care comparison condition is 0.4, that the type I error rate is 5%, and that the correlation between observations on the same subject is 0.5.

### Data Safety and Monitoring Board

The study team has constituted a DSMB including several leading investigators. DSMB members were recruited because of expertise covering behavioral intervention development, global mental health, and implementation. The DSMB meets at least annually and is provided with a report on study progress and enrollment and details of all adverse events. The DSMB includes open and closed sessions.

### Ethical Considerations

The trial was approved by the Human Research Ethics Committees (HREC) of the University of Cape Town (HREC 010/2014) and the Institutional Review Board (IRB) of the University of Miami (IRB Study Number: 20150399). The City of Cape Town research office also approved the study (6584/10530). The trial is registered with ClinicalTrials.gov. Informed consent is obtained from all potential participants before enrollment. Prospective participants are informed of all foreseeable risks of study involvement, that participation is voluntary and does not affect their clinical care, and that they may withdraw their consent without this affecting their medical care. The consent forms are available in English and isiXhosa, the main languages spoken in these clinics. To ensure confidentiality, a unique participant identification number is assigned to participants, and this deidentified number is used on all data collection forms. We collect data using the REDCap electronic data collection platform that transmits data in real time to a central secure database, enhancing data safety. All data not stored electronically, such as copies of consent documents, are stored in locked filing cabinets in designated locked offices. Forms with personal identifying information are stored separately from case report forms. Study records are not available to participants’ health care providers unless requested and documented that participants give consent to this release. We, however, ask participants’ consent to obtain relevant medical information including HIV RNA viral load test results from their clinic folders.

The main risk associated with this study is the initial worsening of symptoms and risk of suicide arising from issues uncovered during counseling [54]. To minimize this potential harm, we train staff to screen all participants who report or display signs of distress for risk of suicide and to provide referrals (based on severity of risk) to appropriate services. These cases are discussed with the study clinicians. There are also potential benefits to participation. All participants benefit from screening for MDD and referral to mental health care as appropriate. We hypothesize that participants in the intervention arm will experience improvements in their mental health, adhere better to ART, and reduce their risk of treatment failure.

## Results

The trial commenced in August 2015 and recruitment began in July 2016. To date, we have screened over 900 participants and randomized 139 participants. A total of 45 participants have exited the study at 12 months. Recruitment continued till July 2019, with the final participant exiting 12 months later in June 2020.

## Discussion

The development of interventions to improve HIV outcomes in the care cascade has become critical to the success of global ART programs. More specifically, attainment of the 90-90-90 The Joint United Nations Programme on HIV/AIDS goals will require a concerted effort, across testing, care engagement, and effective ART adherence. Individuals enrolled onto ART may struggle to adhere for various reasons, but the detection and treatment of depressive illness is a major treatment gap, with potential for significant health and health-economic benefits. In addition, behavioral interventions, especially in LMIC, must be scalable and cost-effective. Scalable means that the intervention can be integrated into primary health care and delivered by nonmental health specialist providers (where professional psychiatrists and psychologists are too few). Cost-effective means that there needs to be a clear benefit to the overall health care and economic system to justify directing resources to recruiting, training, and sustaining interventionists.

The proposed study is unique in that it attempts to address several of these key questions. Most notably, the intervention targets both adherence challenges and depressive symptoms simultaneously. This patient-level integration of ART medication adherence and depression is key to the success of the program. Although the relationship between mental disorder (specifically MDD) and poor adherence is clear, there are few randomized controlled trials demonstrating clear benefits of behavioral interventions on biological outcomes in LMIC. Furthermore, the intervention is being delivered by clinical nurse practitioners with experience in mental health. Nurses are central figures in the growth and development of the South African health care system and, indeed, many LMIC primary health care programs. This is evidenced by the South African Nurse-Initiated Management of Antiretroviral Treatment program [55].

The inclusion of both behavioral and biological outcomes is a major strength of this study. These combined outcomes strengthen the case for improving mental health care services, which have been historically underfunded in many LMIC. Recent data suggest that electronic medication adherence tools (such as Wisepill) are not used uniformly across study samples and, therefore, cannot be used a sole measure of medication use. Successful demonstration of medium- to long-term benefits to sustained viral suppression is key to the efforts in individual- and community-level programs.

It is hoped that a positive outcome in this trial would influence health systems policy not only in South Africa but also in other LMIC. With this in mind, using nurses as interventionists represents a novel approach to complex disorders. Nurses, particularly those with mental health training or experience, may be uniquely suited to fill an important gap in existing task-sharing models, including expanding local capacity for training and supervising nonprofessional providers in CBT and treating more complex, treatment-resistant patients in stepped-care models that also include lay counselors at lower tiers of care. Furthermore, training lay counselors in more complex CBT techniques may be challenging [56] and may not be suitable for more complicated clinical cases. We, therefore, believe that nurses are a provider group uniquely positioned to support scale-up of behavioral interventions to support the needs of more complicated individuals with poor adherence and comorbid psychiatric disorders as well as increase local capacity for training and supervision in CBT in task-sharing models that use lay health workers.

Potential challenges to the success of this trial include difficulties in identifying and recruiting individuals in busy clinic settings, with both virological failure and MDD. To successfully randomize participants, we confirm that the 1-month viral load is >400 copies and that criteria for MDD are met. To address this challenge, we make allowance for additional blood draws in depressed individuals. We work tirelessly with clinic staff in their busy HIV clinics to identify potential participants. A second potential challenge is participant retention through both the acute (intervention) component and the long-term (12 months) outcome period. In this study, PLWH live in periurban areas and may struggle to attend regular planned therapy visits because of casual employment, sickness, or migration. We ensure that we obtain maximal and contemporaneous contact information and work hard to develop rapport with participants across the study. Participants who miss visits are tracked through the clinic system and approached on visit days, and those who migrate may be allowed to complete major assessments by telephone. Hopefully, this will mitigate the risks of attrition. Finally, although previous studies have reported limited or variable success with task-shared behavioral interventions because of poor fidelity, limited skills, or insufficient supervision, major components of this study are intervention supervision (which is performed weekly in a face-to-face fashion), fidelity assessment, and session monitoring. We also note that although we are stratifying for antidepressant prescription across both arms, it is possible that the study itself may result in a change in prescribing behavior. We do not anticipate that this will be a marked effect but will report on this when a final medical record review is conducted at study completion.

In summary, this study has the potential to address several key questions regarding how effective behavioral interventions might be delivered and integrated into primary health care settings in LMIC with high HIV prevalence. Our findings might be used to inform how patients with adherence difficulties and HIV nonsuppression might be effectively treated in primary care, which is critical to motivating to health authorities for scale-up.

## Abbreviations

ART: antiretroviral therapy
ARV: antiretroviral
CBT: cognitive behavioral therapy
CBT-AD: cognitive behavioral therapy intervention for adherence and depression
DSMB: Data Safety and Monitoring Board
ETAU: enhanced treatment as usual
HAM-D: Hamilton Depression Scale
HREC: Human Research Ethics Committees
IA: independent assessor
IRB: Institutional Review Board
LMIC: low- and middle-income countries
MDD: major depressive disorder
MINI: Mini International Neuropsychiatric Interview
NIH: National Institutes of Health
NIMH: National Institute of Mental Health
PLWH: persons living with HIV
RA: research assistant
