# Correction: Nurse-Delivered Cognitive Behavioral Therapy for Adherence and Depression Among People Living With HIV (the Ziphamandla Study): Protocol for a Randomized Controlled Trial

**DOI:** 10.2196/24074

**Published:** 2020-09-28

**Authors:** John A Joska, Lena S Andersen, Rosana Smith-Alvarez, Jessica Magidson, Jasper S Lee, Conall O'Cleirigh, Steven A Safren

**Affiliations:** 1 HIV Mental Health Research Unit, Neuroscience Institute Department of Psychiatry and Mental Health University of Cape Town Cape Town South Africa; 2 Department of Psychology University of Miami Coral Gables, FL United States; 3 Department of Psychology University of Maryland College Park, MD United States; 4 Massachusetts General Hospital, Harvard Medical School Boston, MA United States

In “Nurse-Delivered Cognitive Behavioral Therapy for Adherence and Depression Among People Living With HIV (the Ziphamandla Study): Protocol for a Randomized Controlled Trial” (JMIR Res Protoc 2020;9(2):e14200) the authors noted one error.

Under the Methods section subheading "Secondary Outcomes: HIV Viral Load and CD4 Cell Count," the lower limit of the range of detection for the COBAS AmpliPrep/TaqMan HIV-1 test was reported incorrectly as 48. The actual lower limit of this test is 20. The original text reporting this value read:

Participants who do not have viral load test results within 1 month of the baseline assessment or 4-, 8-, and 12-month follow-up undergo a blood draw for assay using the COBAS AmpliPrep/TaqMan HIV-1 test (range: 48-10,000,000 copies/mL) [50].

This sentence has been corrected to read:

Participants who do not have viral load test results within 1 month of the baseline assessment or 4-, 8-, and 12-month follow-up undergo a blood draw for assay using the COBAS AmpliPrep/TaqMan HIV-1 test (range: 20-10,000,000 copies/mL) [50].

This change does not affect the overall findings of the paper.

The correction will appear in the online version of the paper on the JMIR Publications website on September 28, 2020, together with the publication of this correction notice. Because this was made after submission to PubMed, PubMed Central, and other full-text repositories, the corrected article has also been resubmitted to those repositories.

